# Hepatoprotection by Naringin Nanoliposomes Against Nickel Toxicity Involves Antioxidant Reinforcement and Modulation of Nrf2, NF-κB, PI3K/mTOR, JAK/STAT, and Apoptotic Pathways

**DOI:** 10.3390/ph19010051

**Published:** 2025-12-25

**Authors:** Hussein Abdelaziz Abdalla, Ekramy M. Elmorsy, Najlaa M. M. Jawad, Nora Hosny, Ahmed S. Shams, Hamada S. Salem, Manal S. Fawzy, Mai A. Salem

**Affiliations:** 1 Department of Basic Medical Sciences, College of Medicine, Taibah University, Madinah 42353, Saudi Arabia; huss166@hotmail.com; 2 Department of Medical Biochemistry, Faculty of Medicine, Mansoura University, Mansoura 35516, Egypt; maisalem@mans.edu.eg; 3 Center for Health Research, Northern Border University, Arar 73213, Saudi Arabia; ekramy.elmorsy@nbu.edu.sa; 4 Department of Forensic Medicine and Clinical Toxicology, Faculty of Medicine, Mansoura University, Mansoura 35516, Egypt; 5 Department of Clinical Nutrition, Faculty of Applied Medical Sciences, King Abdulaziz University, Jeddah 21589, Saudi Arabia; nalsini@kau.edu.sa; 6 Department of Medical Biochemistry and Molecular Biology, Faculty of Medicine, Suez Canal University, Ismailia 41522, Egypt; nora_hosny@med.suez.edu.eg; 7 Department of Human Anatomy and Embryology, Faculty of Medicine, Suez Canal University, Ismailia 41522, Egypt; ashams@umn.edu; 8 Department of Zoology, Faculty of Science, Mansoura University, Mansoura 35516, Egypt; hamada2012@mans.edu.eg

**Keywords:** naringin, nanoliposomes, nickel sulfate, hepatotoxicity, antioxidant defense, bioavailability, oxidative stress, inflammation, apoptosis, Nrf2 pathway

## Abstract

**Background/Objectives:** Nickel exposure is a significant environmental and occupational risk factor associated with the onset and progression of chronic liver diseases due to its capacity to induce persistent oxidative stress, inflammation, and hepatocellular injury. This study aimed to evaluate the enhanced hepatoprotective and antioxidant/anti-inflammatory effects of naringin-loaded nanoliposomes (NRG-NLPs), a novel nanoformulation designed to improve the bioavailability of naringin, a citrus-derived flavonoid phytochemical, against nickel sulfate (NiSO_4_)-induced hepatotoxicity in male Wistar rats. **Methods:** Ninety rats were allocated into six groups (n = 15 each): control, NRG, NRG-NLPs, NiSO_4_, NiSO_4_ + NRG, and NiSO_4_ + NRG-NLPs. Treatments consisted of oral administration of NRG or NRG-NLPs (80 mg/kg/day) and intraperitoneal injections of NiSO_4_ (20 mg/kg/day) for three weeks. Endpoints included assessment of growth performance, serum biochemistry, hepatic antioxidant status, inflammatory mediators, apoptotic gene expression, nickel tissue accumulation, and histopathological and ultrastructural liver changes. **Results:** NiSO_4_ exposure induced marked hepatic injury, evidenced by reduced body weight, adverse serum biochemical profiles, increased hepatic enzymes and bilirubin, elevated oxidative damage markers (MDA, protein carbonyls), increased proinflammatory cytokines, and upregulation of HMGB1, PI3K, mTOR, JAK/STAT, and proapoptotic genes, accompanied by aberrant nickel accumulation and severe histopathological alterations. Co-treatment with NRG-NLPs significantly ameliorated biochemical and histological disturbances, restored antioxidant defense systems (SOD, CAT, GPx, GSH, Nrf2, HO-1), and modulated key pathways of inflammation (NF-κB, TNF-α, IL-6), fibrosis (TGF-β), cell survival, and apoptosis more effectively than crude naringin. NRG-NLPs also substantially reduced hepatic nickel deposition and preserved near-normal liver architecture. **Conclusions:** These findings demonstrate that nanoformulated naringin confers superior hepatoprotective benefits against nickel-induced liver injury through enhanced bioavailability and multi-pathway modulation, supporting its translational potential as a citrus-derived medicinal phytochemical and dietary bioactive for the prevention and therapeutic intervention of oxidative and inflammatory chronic liver disease.

## 1. Introduction

Nickel (Ni) is a widely utilized industrial metal and a significant environmental pollutant, with prominent applications in battery production, electroplating, stainless steel fabrication, and alloy manufacturing [1,2]. Human exposure to nickel primarily occurs via inhalation; however, gastrointestinal and dermal absorption also contribute [3]. Following systemic uptake, nickel distributes throughout the body and is mainly excreted renally, with the liver and kidneys acting as principal organs for accumulation and detoxification [1,4]. Consequently, these organs are primary targets for Ni-induced toxicity.

Both experimental and epidemiological studies have demonstrated that chronic exposure to Ni compounds leads to oxidative imbalance, disruption of cellular architecture, and contributes to the development and progression of chronic liver diseases [5,6]. Although the precise mechanisms underlying nickel toxicity remain incompletely characterized, mounting evidence implicates oxidative stress, driven by excess reactive oxygen species (ROS) and depleted antioxidant reserves, notably glutathione, as a central mediator of cellular and tissue injury [7]. ROS-driven disturbances initiate cascades of cellular damage, including lipid peroxidation, protein and DNA oxidation, inflammation, and apoptosis, culminating in organ dysfunction [8]. Additionally, Ni perturbs calcium and thiol homeostasis and promotes free radical formation through redox cycling, further amplifying oxidative stress and cytotoxicity [9]. Prolonged nickel exposure is also implicated in immunosuppression and carcinogenesis in both humans and animal models [3].

Flavonoids, a class of naturally occurring polyphenolic compounds widely distributed in medicinal plants, fruits, vegetables, and other plant-derived foods, exhibit potent antioxidant, anti-inflammatory, and cytoprotective properties due to their capacity to scavenge ROS and modulate intracellular redox status [10]. Naringin (4′,5,7-trihydroxyflavanone-7-rhamnoglucoside), a major flavanone glycoside and key phytoconstituent of grapefruit and other citrus fruits, is a well-characterized medicinal citrus flavonoid that undergoes enzymatic conversion to its aglycone form, naringenin, which mediates its bioactivity [11,12]. Both naringin and naringenin exhibit a wide spectrum of pharmacological effects, including antioxidant, anti-inflammatory, anti-apoptotic, anti-mutagenic, anticancer, lipid-lowering, and hepatoprotective activities [13]. These compounds mitigate oxidative damage by limiting lipid peroxidation, reinforcing antioxidant defenses, and reducing carbonyl protein accumulation [14].

Despite their therapeutic promise, naringin’s poor water solubility and limited oral bioavailability restrict its clinical application [15]. Nanoliposome-based delivery systems have emerged as innovative strategies to enhance the solubility, stability, controlled release, and tissue targeting of plant-derived bioactive molecules [16]. Nanoliposomes are lipid-based nanocarriers that can encapsulate hydrophobic compounds, thereby improving their pharmacokinetic properties and organ specificity, particularly for hepatic delivery [17].

Given the central role of oxidative stress in Ni-induced liver injury and the potent antioxidant activities of naringin, the development of naringin-loaded nanoliposomes represents a promising dietary bioactive for hepatoprotection and chronic disease mitigation. Therefore, the present study aimed to evaluate the protective efficacy of naringin-loaded nanoliposomes (NRG-NLPs) against nickel-induced hepatotoxicity in male rats, compared with crude naringin (NRG). This was achieved by assessing biochemical indices, oxidative and inflammatory mediators, apoptotic markers, and histopathological alterations to elucidate the underlying mechanisms by which this citrus-derived phytochemical formulation mitigates nickel-induced oxidative and inflammatory liver injury.

## 2. Result

### 2.1. Characterization of Naringin-Loaded Nanoliposomes (NRG-NLPs)

**Figure 1:**
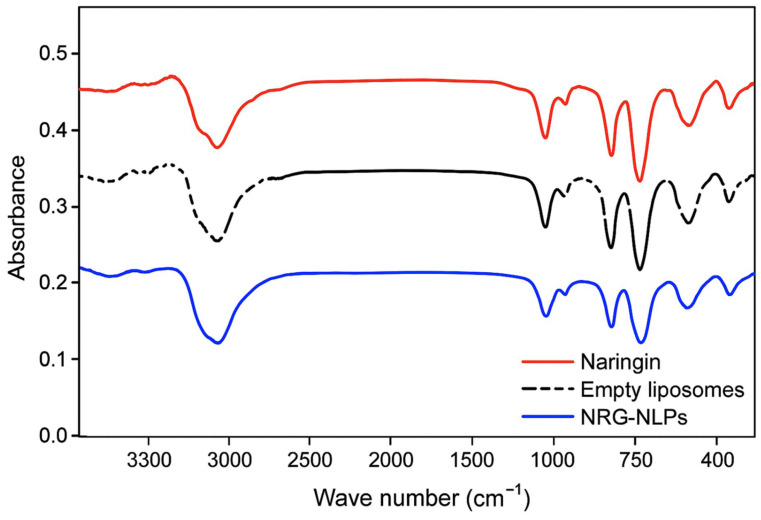
Comparative Fourier Transform Infrared Spectroscopy (FTIR) spectra of pure Naringin, blank liposomes, and Naringin-loaded nanoliposomes (NRG-NLPs).

Figure 1 shows the comparative FTIR spectra of pure Naringin, blank liposomes, and NRG-NLPs. Characteristic peak shifts and band broadening were observed in NRG-NLPs, indicating successful encapsulation and interaction with the lipid matrix.

**Figure 2:**
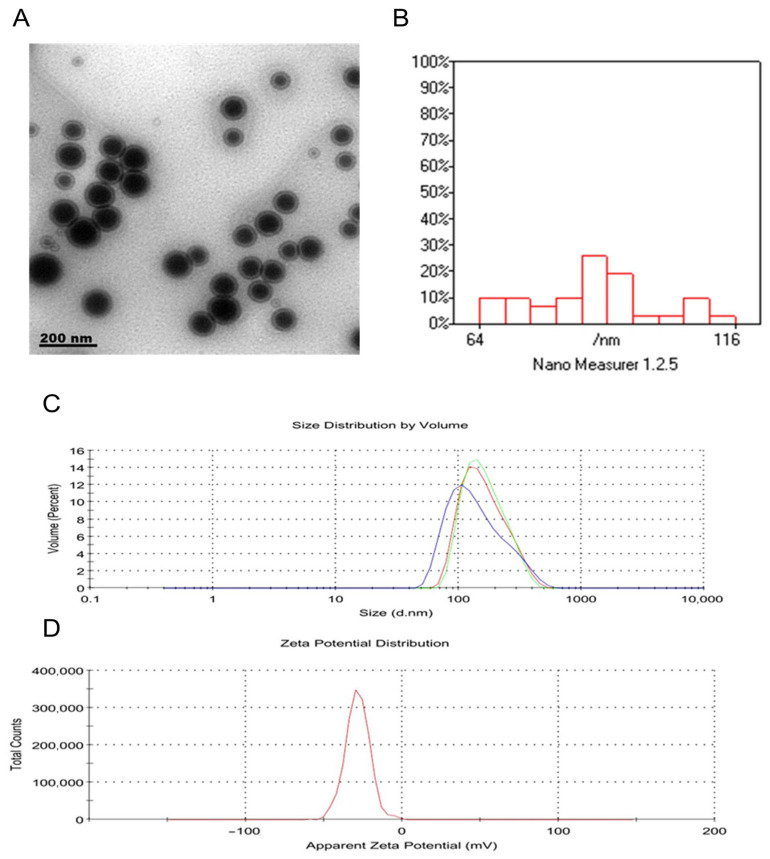
Morphological and physicochemical characterization of naringin-loaded nanoliposomes (NRG-NLPs). (**A**) The morphology of Naringin-Loaded Nanoliposomes by TEM showing nearly spherical particles; (**B**) histogram shows that most particles cluster around 64 to 116 nm, with a good distribution; (**C**) Zeta size distribution by intensity; (**D**) Zeta potential distribution. The values of Z-average size, PDI, and ZP of Naringin-Loaded Nanoliposomes were found to be 164 nm, 0.168, and −28 mV, respectively.

Transmission electron microscopy micrographs revealed that NRG-NLPs were well-dispersed, spherical, and uniform in morphology with no observable aggregation (Figure 2A). The measured particle diameters ranged from 64 to 116 nm (Figure 2B), consistent with optimal nanoscale properties favorable for cellular uptake. Dynamic light scattering analysis indicated that the NRG-NLPs had an average hydrodynamic diameter of 164 nm and a PDI of 0.168, reflecting a moderately homogeneous population (Figure 2C). Zeta potential measurements (Figure 2D) showed a value of −28 mV, supporting colloidal stability through adequate surface charge repulsion. The high encapsulation efficiency (83.93%) confirmed the successful incorporation of naringin into the phospholipid vesicles, contributing to improved physicochemical stability and an anticipated enhancement in bioavailability compared to unencapsulated naringin.

### 2.2. Growth, Food and Water Intake, and Liver Weight

Exposure to nickel sulfate (NiSO_4_) significantly impaired growth and consumption parameters in rats. Ni-treated rats exhibited marked reductions in final body weight, food intake, and water consumption relative to control groups, along with a significant increase in relative liver weight, indicative of systemic and hepatic toxicity. Co-administration of NRG-NLPs resulted in significant improvement of body weight, normalization of food and water intake, and attenuation of liver enlargement versus NiSO_4_-only animals. These observations demonstrate that naringin, particularly when delivered in nanoliposomal form, effectively counteracts Ni-induced toxicity in a dose-dependent manner (Table 1).

### 2.3. Liver Function and Metabolic Parameters

As shown in Table 2, intraperitoneal administration of NiSO_4_ for 21 days produced significant alterations in the serum biochemical profiles of rats compared to all control groups. Levels of total protein, albumin, and globulin were significantly decreased in the NiSO_4_-treated group, indicating impaired hepatic synthetic function. Co-treatment with NRG-NLPs markedly restored these parameters toward normal values, with no significant difference observed compared to the negative control group.

Regarding hepatic function enzymes, significant elevations in ALT, AST, ALP, and total bilirubin were observed in the NiSO_4_ group compared with all controls, consistent with hepatic dysfunction. NRG-NLPs administration significantly reduced the activity of these enzymes, with ALT, AST, and total bilirubin values in the NiSO_4_ + NRG-NLPs group comparable to those of the negative control.

With respect to metabolic parameters, NiSO_4_ exposure induced hyperglycemia and dyslipidemia in experimental rats. Treatment with NRG-NLPs significantly ameliorated these alterations, normalizing glucose and lipid profile values. The improvement was more pronounced in rats treated with NRG-NLPs compared to those receiving crude NRG, demonstrating the superior effectiveness of the nanoformulation in restoring metabolic homeostasis following nickel-induced toxicity.

### 2.4. Hepatic Oxidative Damage

**Figure 3:**
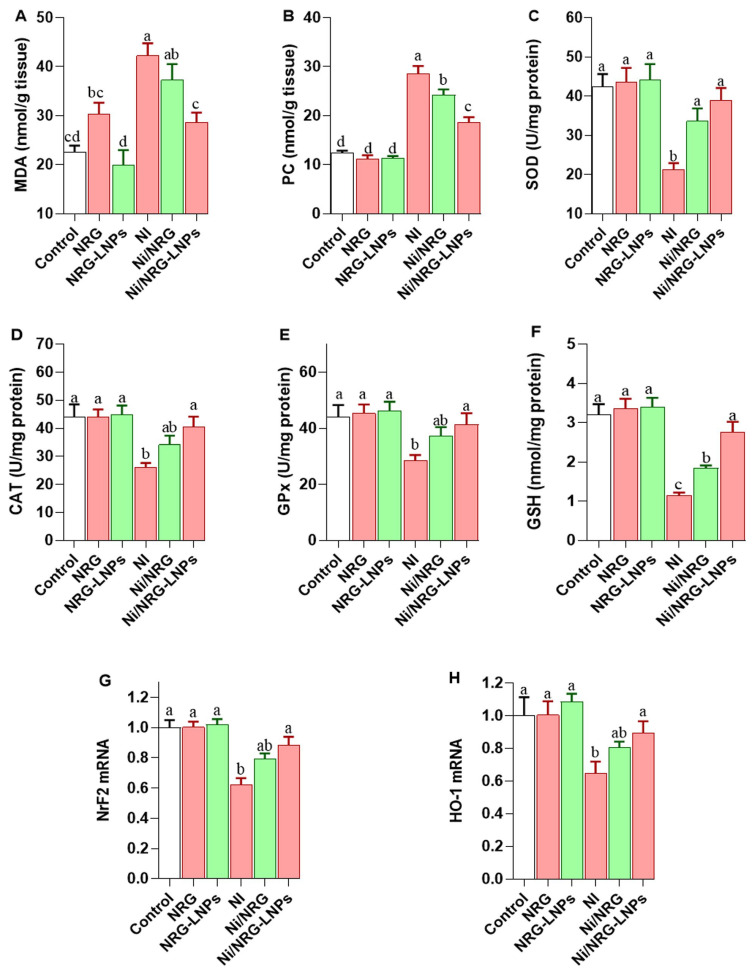
Comparative evaluation of raw and nanoliposomal Naringin on hepatic redox status following nickel sulfate exposure in rats. (**A**) MDA: Malondialdehyde; (**B**) PC: Protein carbonyl; (**C**) SOD: Superoxide dismutase; (**D**) CAT: Catalase; (**E**) GPX: Glutathione peroxidase; (**F**) GSH: Reduced glutathione. (**G**) NrF2 transcript expression; (**H**) HO-1 transcript expression. Group abbreviations: “NRG: Naringin (80 mg/kg body weight); NRG-NLPs: Naringin-loaded liposomal nanoparticles (80 mg/kg body weight); Ni: Nickel sulfate (20 mg/kg body weight); Ni + NRG: Combined treatment of naringin (80 mg/kg body weight) and nickel sulfate (20 mg/kg body weight); Ni + NRG-NLPs: Combined treatment of naringin-loaded liposomal nanoparticles (80 mg/kg body weight) and nickel sulfate (20 mg/kg body weight) (each group n = 10)”. Values are presented as mean ± SE. Significant differences (*p* < 0.05) within the same row are indicated by different superscript letters (a, b, c, d).

Administration of NiSO_4_ significantly elevated hepatic oxidative stress, as demonstrated by increased levels of lipid peroxidation (malondialdehyde, MDA; Figure 3A) and protein oxidation (protein carbonyl, PC; Figure 3B) compared to control groups. Co-treatment with naringin (NRG) or naringin-loaded nanoliposomes (NRG-NLPs) significantly mitigated these alterations, with the greatest protective effect observed in the NRG-NLPs group, indicating enhanced antioxidant capacity and cellular protection conferred by the nanoformulation.

Nickel exposure also led to suppression of key enzymatic antioxidants, including superoxide dismutase (SOD, Figure 3C), catalase (CAT, Figure 3D), and glutathione peroxidase (GPx, Figure 3E), along with reduced levels of reduced glutathione (GSH, Figure 3F) and decreased mRNA expression of Nrf2 and HO-1 (Figure 3G,H), reflecting impaired hepatic redox homeostasis. Importantly, administration of NRG-NLPs efficiently restored these antioxidant parameters to near-normal levels, with no significant difference compared with negative controls. These results confirm that nanoliposomal delivery of NRG significantly enhances its bioavailability and efficacy in counteracting Ni-induced oxidative stress by reinforcing endogenous antioxidant defense mechanisms.

### 2.5. Hepatic Inflammatory Response

**Figure 4:**
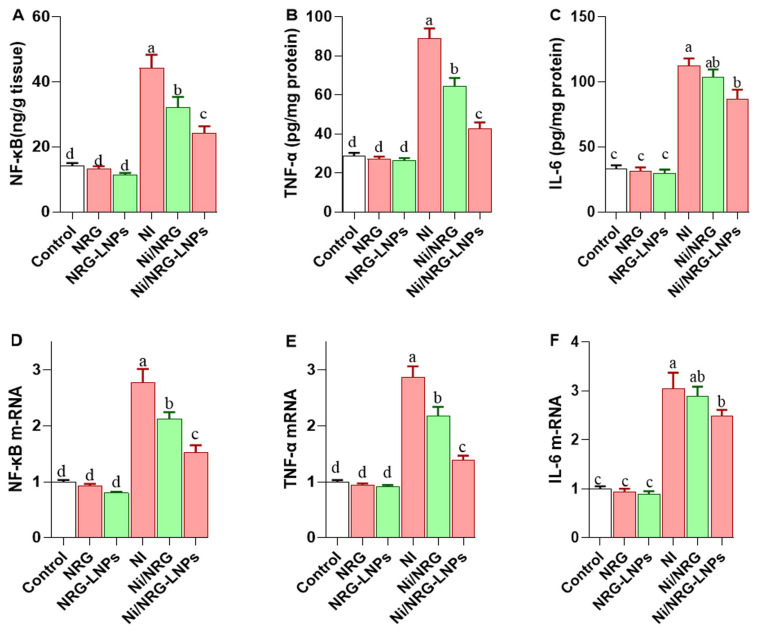
Comparative evaluation of raw and nanoliposomal naringin on hepatic inflammatory response following nickel sulfate exposure in rats. (**A**) NF-κB: Nuclear Factor kappa-light-chain-enhancer of activated B cells; (**B**) TNF-α: Tumor Necrosis Factor-alpha; (**C**) IL-6: Interleukin-6. (**D**) NF-κB transcript expression; (**E**) TNF-α transcript expression; (**F**) IL-6 transcript expression. Group abbreviations: “NRG: Naringin (80 mg/kg body weight); NRG-NLPs: Naringin-loaded liposomal nanoparticles (80 mg/kg body weight); Ni: Nickel sulfate (20 mg/kg body weight); Ni + NRG: Combined treatment of naringin (80 mg/kg body weight) and nickel sulfate (20 mg/kg body weight); Ni + NRG-NLPs: Combined treatment of naringin-loaded liposomal nanoparticles (80 mg/kg body weight) and nickel sulfate (20 mg/kg body weight (each group n = 10)”. Values are presented as mean ± SE. Significant differences (*p* < 0.05) within the same row are indicated by different superscript letters (a, b, c, d).

Exposure to NiSO_4_ markedly intensified the hepatic inflammatory response, as indicated by significant increases in nuclear factor kappa B (NF-κB; Figure 4A), tumor necrosis factor-alpha (TNF-α; Figure 4B), and interleukin-6 (IL-6; Figure 4C) levels, along with upregulation of their respective coding genes (Figure 4D–F), compared to all control groups. Co-treatment with naringin (NRG) or its nanoliposomal formulation (NRG-NLPs) effectively attenuated these inflammatory changes, with the most pronounced reductions observed in the NRG-NLPs group. Although IL-6 levels were significantly elevated in the NiSO_4_ group, administration of NRG-NLPs significantly reduced these levels. No significant difference in IL-6 was observed between the NiSO_4_ and NiSO_4_ + NRG groups, highlighting the superior anti-inflammatory effect of the nanoformulation over crude NRG in mitigating nickel-induced hepatic inflammation.

### 2.6. Hepatic TGF-β Levels

**Figure 5:**
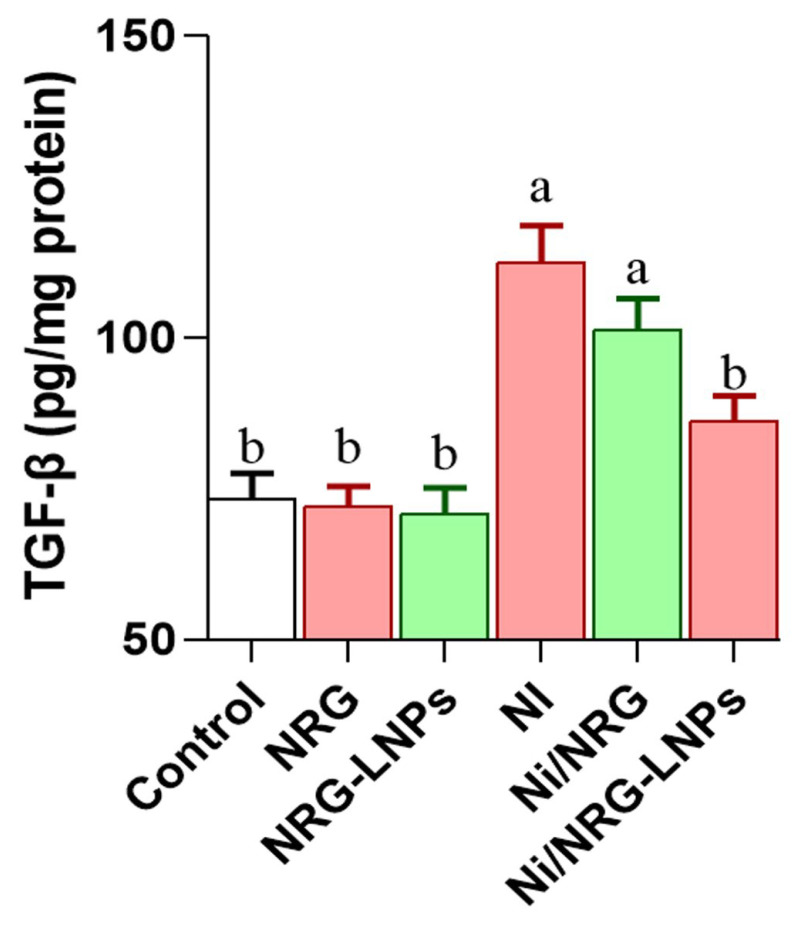
Comparative evaluation of raw and nanoliposomal Naringin on hepatic transforming growth factor-beta (TGF-β) levels following nickel sulfate exposure in rats. Group abbreviations: “NRG: Naringin (80 mg/kg body weight); NRG-NLPs: Naringin-loaded liposomal nanoparticles (80 mg/kg body weight); Ni: Nickel sulfate (20 mg/kg body weight); Ni + NRG: Combined treatment of naringin (80 mg/kg body weight) and nickel sulfate (20 mg/kg body weight); Ni + NRG-NLPs: Combined treatment of naringin-loaded liposomal nanoparticles (80 mg/kg body weight) and nickel sulfate (20 mg/kg body weight) (each group n = 10)”. Values are presented as mean ± SE. Significant differences (*p* < 0.05) within the same row are indicated by different superscript letters (a, b).

Nickel sulfate (NiSO_4_) exposure resulted in a significant elevation of hepatic TGF-β concentrations compared to all control groups (Figure 5), indicative of activation of fibrogenic and inflammatory signaling pathways. Co-treatment with naringin-loaded nanoliposomes (NRG-NLPs) significantly reduced TGF-β levels, demonstrating a superior inhibitory effect compared to crude naringin (NRG). Notably, there was no significant difference in TGF-β levels between the NiSO_4_-only and NiSO_4_ + NRG groups, underscoring the enhanced anti-fibrotic efficacy of the nanoformulated NRG.

### 2.7. Hepatic HMGB1, PI3K, and mTOR Protein Levels

**Figure 6:**
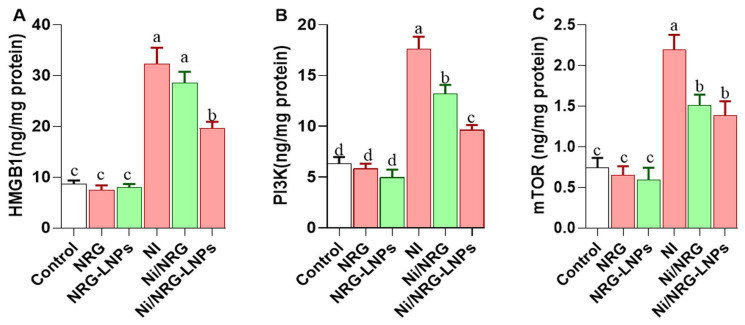
Comparative evaluation of raw and nanoliposomal naringin on hepatic HMGB1, PI3K, and mTOR protein levels following nickel sulfate exposure in rats. (**A**) HMGB1: High Mobility Group Box 1; (**B**) PI3K: Phosphoinositide 3-kinase; (**C**) mTOR: Mammalian Target of Rapamycin. Group abbreviations: “NRG: Naringin (80 mg/kg body weight); NRG-NLPs: Naringin-loaded liposomal nanoparticles (80 mg/kg body weight); Ni: Nickel sulfate (20 mg/kg body weight); Ni + NRG: Combined treatment of naringin (80 mg/kg body weight) and nickel sulfate (20 mg/kg body weight); Ni + NRG-NLPs: Combined treatment of naringin-loaded liposomal nanoparticles (80 mg/kg body weight) and nickel sulfate (20 mg/kg body weight) (each group n = 10)”. Values are presented as mean ± SE. Significant differences (*p* < 0.05) within the same row are indicated by different superscript letters (a, b, c, d).

As shown in Figure 6, ELISA analysis revealed significant increases in hepatic high-mobility group box 1 (HMGB1), phosphoinositide 3-kinase (PI3K), and mechanistic target of rapamycin (mTOR) protein levels in the NiSO_4_-treated group compared to all control groups, indicating the activation of inflammatory and prosurvival pathways. Co-treatment with naringin-loaded nanoliposomes (NRG-NLPs) significantly reduced HMGB1 and PI3K levels, demonstrating a stronger suppressive effect than crude naringin (NRG). For mTOR, no significant difference was observed between NRG and NRG-NLP treatments, suggesting that both formulations have a comparable modulatory effect on mTOR signaling.

### 2.8. Hepatic JAK/STAT Signaling Pathway

**Figure 7:**
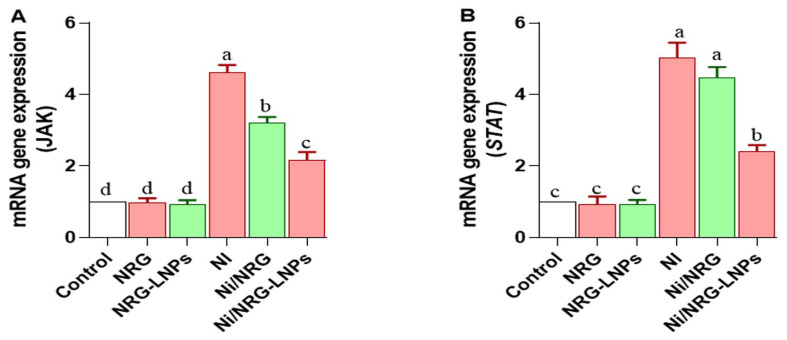
Comparative evaluation of raw and nanoliposomal naringin on hepatic *JAK/STAT* signaling pathway following nickel sulfate exposure in rats. (**A**) *JAK*: Janus Kinase; (**B**) STAT: Signal Transducer and Activator of Transcription. Group abbreviations: “NRG: Naringin (80 mg/kg body weight); NRG-NLPs: Naringin-loaded liposomal nanoparticles (80 mg/kg body weight); Ni: Nickel sulfate (20 mg/kg body weight); Ni + NRG: Combined treatment of naringin (80 mg/kg body weight) and nickel sulfate (20 mg/kg body weight); Ni + NRG-NLPs: Combined treatment of naringin-loaded liposomal nanoparticles (80 mg/kg body weight) and nickel sulfate (20 mg/kg body weight) (each group n = 10)”. Values are presented as mean ± SE. Significant differences (*p* < 0.05) within the same row are indicated by different superscript letters (a, b, c, d).

NiSO_4_ exposure significantly upregulated hepatic *JAK* (Figure 7A) and *STAT* (Figure 7B) expression compared with all control groups, indicating activation of the JAK/STAT signaling pathway in response to nickel-induced stress. Co-treatment with NRG-NLPs significantly suppressed the upregulation of both *JAK* and *STAT*, demonstrating a superior modulatory effect compared to crude NRG. No significant difference was observed between the NiSO_4_ and NiSO_4_ + NRG groups, underscoring the enhanced inhibitory potential of the nanoformulated NRG against this proinflammatory signaling cascade.

### 2.9. Apoptotic Gene Expression Profile

**Figure 8:**
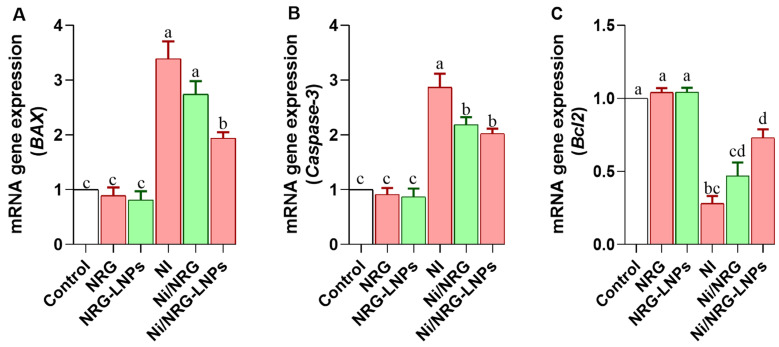
Comparative evaluation of raw and nanoliposomal naringin on apoptotic gene expression profile following nickel sulfate exposure in rats. (**A**) *Bax*: Bcl-2-associated X protein; (**B**) *Caspase-3*: Cysteine-aspartic acid protease-3; (**C**) *Bcl2*: B-cell lymphoma 2. Group abbreviations: “NRG: Naringin (80 mg/kg body weight); NRG-NLPs: Naringin-loaded liposomal nanoparticles (80 mg/kg body weight); Ni: Nickel sulfate (20 mg/kg body weight); Ni + NRG: Combined treatment of naringin (80 mg/kg body weight) and nickel sulfate (20 mg/kg body weight); Ni + NRG-NLPs: Combined treatment of naringin-loaded liposomal nanoparticles (80 mg/kg body weight) and nickel sulfate (20 mg/kg body weight) (each group n = 10)”. Values are presented as mean ± SE. Significant differences (*p* < 0.05) within the same row are indicated by different superscript letters (a, b, c, d).

NiSO_4_ exposure elicited a pronounced proapoptotic response in hepatic tissue, evidenced by significant upregulation of *Bax* (Figure 8A) and *Caspase-3* (Figure 8B) gene expression, along with marked downregulation of the anti-apoptotic gene *Bcl-2* (Figure 8C). Co-treatment with NRG-NLPs effectively restored the apoptotic balance, downregulating *Bax* and *Caspase-3* while upregulating *Bcl-2*, indicating the potent anti-apoptotic and cytoprotective activity of the nanoliposomal NRG formulation against nickel-induced hepatocellular injury.

### 2.10. Hepatic Nickel Accumulation

**Figure 9:**
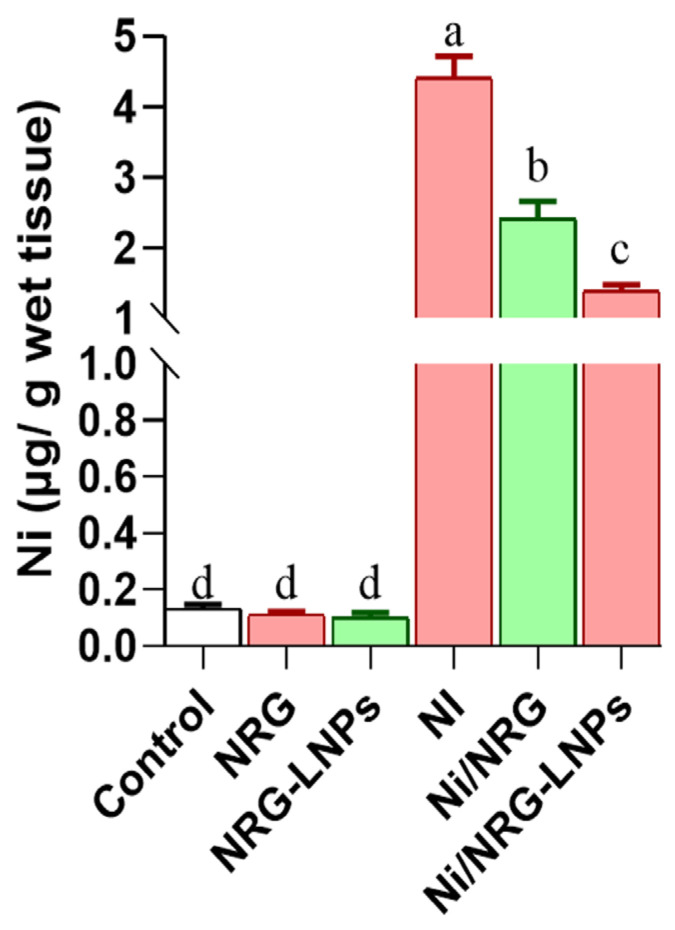
Comparative evaluation of raw and nanoliposomal naringin on hepatic nickel (Ni) accumulation following nickel sulfate exposure in rats. Group abbreviations: “NRG: Naringin (80 mg/kg body weight); NRG-NLPs: Naringin-loaded liposomal nanoparticles (80 mg/kg body weight); Ni: Nickel sulfate (20 mg/kg body weight); Ni + NRG: Combined treatment of naringin (80 mg/kg body weight) and nickel sulfate (20 mg/kg body weight); Ni + NRG-NLPs: Combined treatment of naringin-loaded liposomal nanoparticles (80 mg/kg body weight) and nickel sulfate (20 mg/kg body weight) (each group n = 10)”. Values are presented as mean ± SE. Significant differences (*p* < 0.05) within the same row are indicated by different superscript letters (a, b, c, d).

NiSO_4_ exposure led to a significant hepatic accumulation of Ni, as shown in Figure 9, indicating effective metal uptake and retention in liver tissue. Rats treated with NiSO_4_ exhibited markedly elevated hepatic nickel concentrations compared to all control groups, reflecting heightened tissue susceptibility to metal deposition. Co-administration of NRG or NRG-NLPs significantly reduced the degree of nickel accumulation, suggesting a capacity to chelate or counteract nickel uptake. Notably, the NRG-NLPs group demonstrated the lowest hepatic nickel concentration among the treatment groups, underscoring the nanoformulation’s superior protective and detoxifying efficacy in reducing hepatic metal burden.

### 2.11. Hepatic Histopathological Alterations

**Figure 10:**
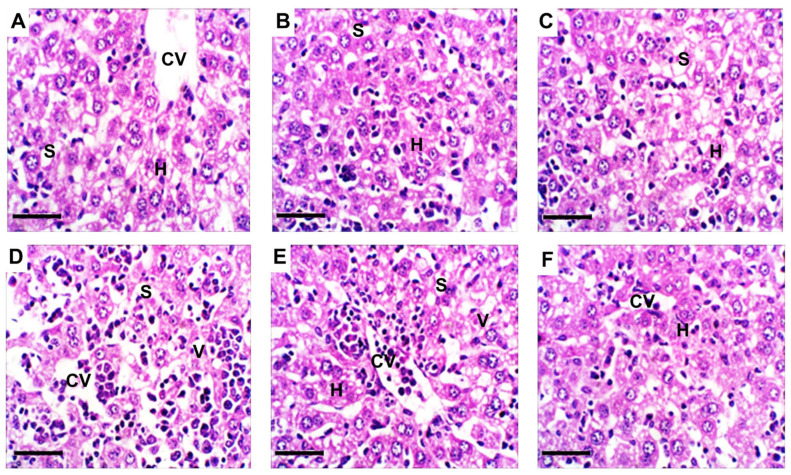
Representative photomicrographs of liver sections from control and experimental groups under heat stress: (**A**) control group; (**B**) NRG group; (**C**) NRG-NLPs group; (**D**) Ni group; (**E**) Ni + NRG group; and (**F**) Ni + NRG-NLPs group. Structural features include the central vein (CV), hepatocytes (H), and sinusoids (S). Cytoplasmic vacuolation (V) is indicated where present. All images were captured at 400× magnification; scale bar = 50 μm.

**Figure 11:**
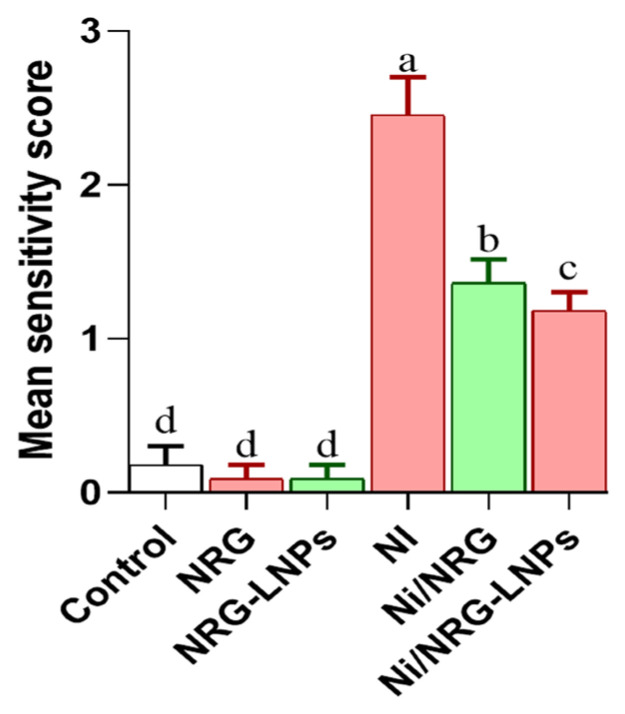
Severity scores of liver tissue damage in the different experimental groups following nickel sulfate exposure in rats. Group abbreviations: “NRG: Naringin (80 mg/kg body weight); NRG-NLPs: Naringin-loaded liposomal nanoparticles (80 mg/kg body weight); Ni: Nickel sulfate (20 mg/kg body weight); Ni + NRG: Combined treatment of naringin (80 mg/kg body weight) and nickel sulfate (20 mg/kg body weight); Ni + NRG-NLPs: Combined treatment of naringin-loaded liposomal nanoparticles (80 mg/kg body weight) and nickel sulfate (20 mg/kg body weight) (each group n = 10)”. Values are presented as mean ± SE. Significant differences (*p* < 0.05) within the same row are indicated by different superscript letters (a, b, c, d).

Microscopic analysis showed that NRG and NRG-NLPs did not induce detectable histological changes; lobular architecture, central veins, and hepatocyte morphology were preserved (Figure 10A–C). NiSO_4_-exposed livers exhibited pronounced lesions, including central vein congestion, hepatocellular vacuolation, and necrosis (Figure 10D). Both NRG and NRG-NLPs treatments markedly counteracted these alterations, restoring hepatocyte cords and promoting recovery of normal lobular structure (Figure 10E,F). NiSO_4_ exposure resulted in a notable increase in hepatic lesion scores, whereas concurrent treatment with NRG or NRG-NLPs substantially reduced these scores, confirming their restorative effect on liver architecture (Figure 11).

### 2.12. Hepatic Ultrastructural Alterations

**Figure 12:**
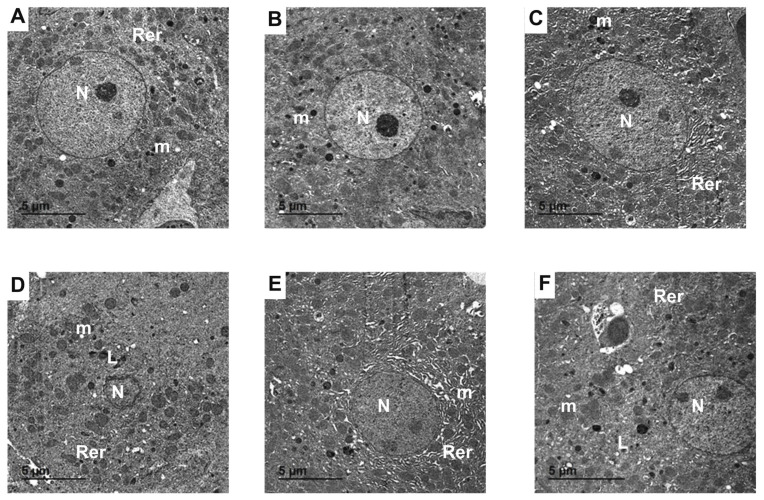
Representative transmission electron micrographs showing hepatic ultrastructure from the control and experimental groups: (**A**) control group; (**B**) NRG group; (**C**) NRG-NLPs group; (**D**) Ni group; (**E**) Ni + NRG group; and (**F**) Ni + NRG-NLPs group. Key ultrastructural components are indicated, including the nucleus (N), mitochondria (m), rough endoplasmic reticulum (Rer), and lysosomes (L).

Ultrastructural analysis demonstrated that hepatocytes from the control (Figure 12A), NRG (Figure 12B), and NRG-NLPs (Figure 12C) groups retained normal morphology: spherical nuclei with visible nucleoli, organized rough endoplasmic reticulum, and mitochondria with intact membranes and cristae. In NiSO_4_-treated livers, TEM revealed pronounced necrotic changes, including extensive vacuolation, dilated rough endoplasmic reticulum, chromatin condensation, mitochondrial swelling with fragmented cristae, and irregular nuclei (Figure 12D). Concurrent administration of NRG or NRG-NLPs with Ni markedly restored normal hepatocellular ultrastructure. Hepatocytes displayed well-defined nuclei, intact mitochondria (with only minimal vacuolation or degeneration), and organized endoplasmic reticulum (Figure 12E,F).

## 3. Discussion

Ni exposure has been widely recognized for its toxic potential, capable of inducing diverse physiological, biochemical, and behavioral disorders across species [3]. The liver is a principal target organ for Ni toxicity due to its essential roles in metabolism and detoxification of xenobiotics [18,19]. Prolonged Ni exposure leads to hepatic accumulation, disrupting hepatic lobular architecture and causing extensive hepatocyte injury by activating multiple oxidative and inflammatory signaling cascades [2].

Natural antioxidant compounds are increasingly recognized for their hepatoprotective properties, which can attenuate oxidative stress and maintain redox stability. Such compounds neutralize ROS, limit lipid and protein peroxidation, and downregulate inflammatory mediators implicated in hepatic deterioration [20]. Among these, NRG is known for its multifaceted ability to counteract oxidative and inflammatory damage in the liver. NRG acts through mechanisms that include free radical neutralization, upregulation of antioxidant defenses (GPx, SOD, CAT), and inhibition of proinflammatory signaling pathways such as NF-κB and MAPK, thereby supporting the structural and functional integrity of the liver [11].

Despite its therapeutic efficacy, the clinical application of NRG is limited by unfavorable physicochemical properties, including poor stability, low water solubility, and limited oral bioavailability. To overcome these issues, the present study hypothesized that liposomal encapsulation (NRG-NLPs) could enhance NRG’s solubility, stability, and bioavailability, thereby improving its hepatoprotective capacity. This rationale guided the evaluation of NRG-NLPs for their ability to mitigate Ni-induced hepatic toxicity in rats, compared with unencapsulated NRG.

Body weight is considered a reliable marker for systemic toxicity in animal studies [21]. Here, Ni exposure induced marked reductions in body weight and an increase in liver weight, indicating hepatic stress from metabolic disruption, reduced appetite, or direct hepatocellular injury [22,23]. The tendency for Ni to accumulate in the liver is attributed to high hepatic expression of metallothionein, a metal-binding protein with high affinity for metal ions [24]. The reduction in hepatic Ni concentration following NRG-NLPs administration observed in this study suggests possible metal chelation, facilitating Ni detoxification and elimination.

NRG’s chelating activity is linked to the hydroxyl groups in its structure and metabolites, which form stable complexes with Ni ions, reducing toxicity [13,14]. Consistent with this, Ni exposure resulted in significant disturbances in hepatic function markers (ALT, AST, ALP, TBIL), with increases reflecting hepatocellular membrane disruption and cholestatic impairment [4,25]. Such elevation is indicative of mitochondrial damage and release of cytosolic enzymes [26,27]. Ni exposure also reduced serum protein levels (TP, albumin, globulin), pointing to impaired hepatic protein synthesis.

Remarkably, co-treatment with NRG-NLPs reversed the increases in serum enzyme activities and restored protein concentrations more efficiently than crude NRG. This suggests enhanced preservation of hepatic metabolic and biosynthetic capacity with the nanoformulation. In addition, Ni disrupted serum lipid and glucose profiles (hyperglycemia and dyslipidemia), hallmark features of hepatic dysfunction [28]. NRG-NLPs administration normalized these metabolic disturbances, an effect attributed to its antioxidant properties, restoration of membrane integrity, and stabilization of glucoregulatory hormones [29,30].

A central role of oxidative stress in Ni hepatotoxicity was confirmed by reduced SOD, CAT, GPx, and GSH, and by a marked elevation in MDA and PC, indicative of excessive free radical production and antioxidant breakdown [6,7,23]. NRG’s potent antioxidant activity is attributed to its hydroxyl groups and dual actions: radical scavenging and metal chelation, which preserve non-enzymatic antioxidants and increase levels of vitamins C and E, sulfhydryls, and GSH [31,32,33]. NRG-NLPs further potentiated these effects, resulting in optimal hepatic protection against Ni-induced damage.

Histopathological and ultrastructural analyses supported these functional findings: Ni intoxication induced significant tissue disorganization, congestion, vacuolation, and necrosis. NRG-NLP treatment ameliorated these changes, demonstrating preservation of hepatic architecture with only mild alterations, as observed in other studies using NRG or other natural products to mitigate toxic liver injury [34,35,36,37].

At the molecular signaling level, Ni exposure triggered activation of inflammatory cascades (NF-κB, TNF-α, IL-6, HMGB1, PI3K/mTOR) and apoptotic pathways (JAK/STAT3, Bax, Caspase-3), along with downregulation of anti-apoptotic Bcl-2, linking chronic inflammation to programmed cell death [38,39,40,41,42]. Elevated TGF-β levels further drive hepatic fibrosis and stellate cell activation [43,44,45,46].

NRG-NLPs administration significantly suppressed these pathogenic pathways, reducing oxidative and inflammatory mediators, inhibiting fibrogenic and proapoptotic signaling (JAK/STAT, Bax, Caspase-3), and increasing Bcl-2 expression. Histological and ultrastructural restoration paralleled these molecular corrections.

The current findings align with Mansour et al., who reported anti-inflammatory and anti-apoptotic effects of NRG against lead acetate–induced hepatocyte injury [47]. Specifically, NRG suppressed NF-κB activation, increased IL-6, upregulated Bcl-2, and inhibited caspase-3, thereby protecting hepatocytes from inflammatory and apoptotic damage [48].

**Figure 13:**
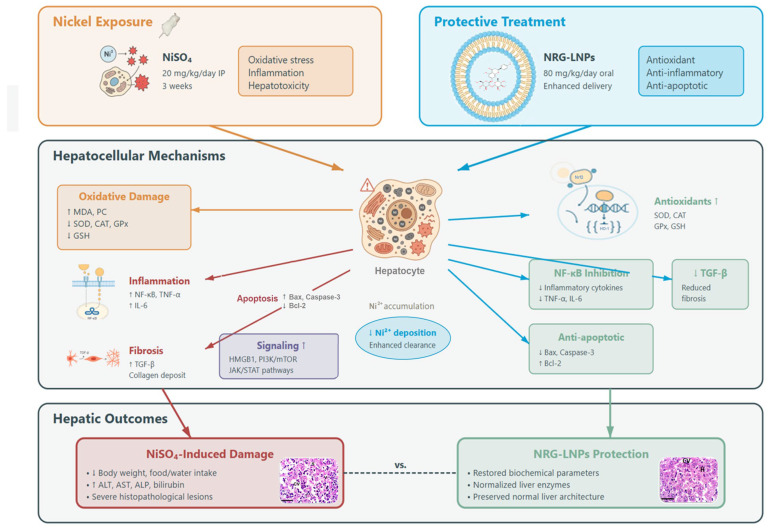
Schematic overview of the hepatoprotective effects of naringin-loaded nanoliposomes (NRG-NLPs) against nickel sulfate (NiSO_4_)-induced liver injury in rats. Chronic NiSO_4_ exposure (20 mg/kg/day, intraperitoneally, for 3 weeks) leads to hepatotoxicity characterized by oxidative stress (↑ MDA, protein carbonyls; ↓ SOD, CAT, GPx, GSH), inflammation (↑ NF-κB activation, TNF-α, IL-6), fibrosis (↑ TGF-β, collagen deposition), dysregulated signaling (↑ HMGB1, PI3K/mTOR, JAK/STAT), apoptosis (↑ Bax, caspase-3; ↓ Bcl-2), Ni^2+^ accumulation in hepatocytes, and adverse hepatic outcomes (↓ body weight and food/water intake; ↑ ALT, AST, ALP, bilirubin; severe histopathological lesions). In contrast, oral administration of NRG-NLPs (80 mg/kg/day) enhances naringin delivery and exerts antioxidant (restoration of SOD, CAT, GPx, GSH, Nrf2/HO-1), anti-inflammatory (NF-κB inhibition; ↓ TNF-α, IL-6), anti-fibrotic (↓ TGF-β), anti-apoptotic (↓ Bax and caspase-3; ↑ Bcl-2), and Ni^2+^-clearing effects, thereby normalizing biochemical parameters, improving liver enzyme profiles, reducing hepatic nickel deposition, and preserving near-normal liver architecture. ↑: Increase; ↓: decrease; vs.: versus. Adapted from GRABSTRACT-Science, visualized (https://grabstract.io/) (accessed 2 December 2025).

Taken together, the results of this study substantiate the multifaceted efficacy of nanoliposomal NRG in mitigating Ni-induced hepatic injury. By exerting robust antioxidant, anti-inflammatory, anti-apoptotic, and anti-fibrotic activities, NRG-NLPs offer a promising approach for the prevention and management of heavy metal-induced liver injury (Figure 13).

### Study Limitations and Future Perspectives

Despite the promising hepatoprotective effects of NRG-NLPs demonstrated in this study, several limitations should be considered. First, the investigation was confined to an acute rat model and may not fully reflect chronic toxicity scenarios or human liver pathophysiology. Second, while molecular and biochemical analyses point to mechanistic actions, further in-depth studies are required to elucidate specific internalization pathways and the biodistribution of NRG-NLPs. Third, only male rats were used; thus, sex-related variations in response cannot be excluded. Fourth, a NiSO_4_ + blank nanoliposomes group was not included, which would further help exclude any potential independent effects of the liposomal carrier. Lastly, long-term safety, optimal dosing, and pharmacokinetics of NRG-NLPs remain to be established. Future studies should address these limitations by extending the experimental duration, including both sexes, and utilizing advanced imaging and biodistribution techniques. Moreover, the application of multi-omics approaches (genomics, proteomics, metabolomics) and probe-based technologies, including traditional probes and the novel PROTAC probe technology, will enable more comprehensive investigation of NRG’s targets and mechanisms of action [49,50]. Investigations into NRG-NLPs’ effects in chronic and multi-organ toxicity models, as well as in clinical translational studies, will be crucial for confirming their utility as a safe and effective intervention against heavy metal–induced organ injury.

## 4. Materials and Methods

### 4.1. Preparation of Naringin-Loaded Nanoliposomes (NRG-NLPs)

Naringin-loaded nanoliposomes were synthesized using the conventional thin-film hydration technique. Briefly, naringin (NRG; 100 mg; Sigma-Aldrich, St. Louis, MO, USA) and soybean lecithin (500 mg) were dissolved in 100 mL of organic solvent. The solution was subjected to rotary evaporation (Heidolph LABOROTA 4000, Heidolph Instruments GmbH & Co. KG, Schwabach, Germany) at 60 °C and 70 rpm under vacuum, yielding a thin lipid film. This film was hydrated with 50 mL of deionized water at 60 °C under constant stirring (120 rpm) for 50 min, yielding a crude nanoliposomal suspension.

To achieve uniform particle size, the suspension was sonicated in a bath sonicator for 40 min, followed by probe sonication in an ice bath (amplitude: 60%; duration: 3 min; pulse: 1 s ON, 1 s OFF). The resulting dispersion was filtered through a 0.45 μm syringe filter and stored at 4 °C until use.

### 4.2. Characterization of Nanoliposomes

#### 4.2.1. Particle Size, PDI, and Zeta Potential

Hydrodynamic mean diameter, polydispersity index (PDI), and zeta potential were measured in triplicate using a Zetasizer NanoZS (Malvern, UK) following appropriate dilution in distilled water.

#### 4.2.2. Morphology

Particle morphology and size distribution were visualized and quantified by transmission electron microscopy (TEM; JEM-2100, JEOL Ltd., Akishima, Tokyo, Japan) operated at 160 kV, analyzing at least 100 nanoparticles per sample for statistical reliability. Micrographs were processed using Digital Micrograph (Gatan Microscopy Suite, Gatan Inc., Pleasanton, CA, USA) and iTEM/SoftImagingSystem software (Olympus Soft Imaging Solutions GmbH, Münster, Germany).

#### 4.2.3. Chemical Integrity and Encapsulation

Fourier-transform infrared (FTIR) spectroscopy (PerkinElmer, Waltham, MA, USA; 4000–400 cm^−1^) was performed on dried samples of NRG, blank liposomes, and NRG-NLPs. Characteristic spectral peaks of NRG and NRG-NLPs were compared to confirm successful encapsulation and assess interactions with the lipid matrix.

#### 4.2.4. Encapsulation Efficiency (LE)

NRG loading efficiency was calculated using a calibration curve (334 nm). After preparation, samples were centrifuged (15,000× *g*, 30 min), and unencapsulated NRG in the supernatant was quantified spectrophotometrically. LE (%) = [encapsulated NRG/NRG added] × 100.

#### 4.2.5. Stability

Physical stability of NRG-NLPs was evaluated by monitoring particle size, PDI, and zeta potential at 0, 15, and 30 days following storage at 4 °C. Measurements were performed in triplicate and expressed as mean ± SD. Zeta potential values exceeding ±25 mV were considered indicative of colloidal stability.

#### 4.2.6. In Vitro Release and Analysis

NRG release from nanoliposomes was assessed using a dialysis membrane method in phosphate-buffered saline (PBS, pH 7.4) at 37 °C with gentle shaking. Aliquots were sampled at predetermined intervals and analyzed spectrophotometrically at 334 nm. All measurements were conducted in triplicate and reported as mean ± SD. Statistical comparisons were performed using one-way ANOVA followed by Tukey’s post hoc test; *p* < 0.05 was considered statistically significant.

### 4.3. Animals and Experimental Protocol

Adult male Wistar rats (n = 90; body weight 176.06 ± 14.93 g) were obtained from the “Medical Experimental Research Center (MERC), Faculty of Medicine, Mansoura University, Egypt.” The animals were housed in standard polypropylene cages (five rats per cage) under controlled conditions (12 h light/dark cycle, 24 ± 1 °C, 45% relative humidity) with ad libitum access to standard rodent chow and water. Rats were allowed a 2-week acclimatization period before the initiation of experimental procedures.

All experimental procedures were carried out following the “National Institutes of Health Guide for the Care and Use of Laboratory Animals (NIH Publication No. 8023, revised 1996)” and were approved by the “Institutional Animal Care and Use Committee (IACUC) of Mansoura University (Approval No. MU-ACUC; SC.R.25.11.37).”

#### 4.3.1. Randomization and Blinding

Rats were randomly assigned to six experimental groups (n = 15 per group) using a computer-generated randomization schedule. Group allocations were concealed from the researchers responsible for biochemical, histopathological, and molecular evaluations, ensuring outcome assessment was performed in a blinded manner.

#### 4.3.2. Sample Size Rationale

The group size was determined based on previous studies with similar models of hepatotoxicity and power analysis [33,51]. Calculations were designed to detect a minimum 25% difference in key biochemical parameters between groups, with a power of 0.8 and a two-sided significance level of 0.05. Ten rats per group were used for endpoint analyses; the remainder served as backups to account for possible mortality or technical exclusions.

#### 4.3.3. Experimental Groups and Treatment Protocols

Following acclimation, rats were treated as follows:

- Group I (Negative control): 0.9% saline (5 mL/kg, orally) once daily for 3 weeks.
- Group II (NRG): Crude naringin (80 mg/kg, suspended in 5 mL of 0.9% saline, orally) once daily for 3 weeks.
- Group III (NRG-NLPs): Naringin-loaded nanoliposomes (80 mg/kg, suspended in 0.9% saline, orally) once daily for 3 weeks.
- Group IV (NiSO_4_): 0.9% saline (5 mL/kg, orally) and nickel sulfate (20 mg/kg body weight, intraperitoneally) once daily for 3 weeks.
- Group V (NiSO_4_ + NRG): Nickel sulfate (20 mg/kg, i.p.) and crude naringin (80 mg/kg, orally) once daily for 3 weeks, with naringin administered 2 h before NiSO_4_ injection.
- Group VI (NiSO_4_ + NRG-NLPs): Nickel sulfate (20 mg/kg, i.p.) and naringin-loaded nanoliposomes (80 mg/kg, orally) once daily for 3 weeks, with NRG-NLPs administered 2 h before NiSO_4_ injection. The selected doses of naringin and nickel sulfate were based on a previous study [33].

In the NRG-NLPs–treated groups, the administered oral dose (80 mg/kg) was calculated based on the amount of encapsulated naringin rather than the total nanoliposomal formulation, and the dosing volume was adjusted to account for the determined encapsulation efficiency to ensure dose equivalence with the crude naringin–treated groups.

#### 4.3.4. Endpoints and Monitoring

Throughout the experimental period, rats were monitored daily for signs of illness, toxicity, or distress, with particular attention to activity, posture, fur condition, and feeding/drinking behavior. Body weight, food intake, and water intake were recorded every 3 days. Humane endpoint criteria were established; animals exhibiting rapid weight loss (>15%), persistent lethargy, severe dehydration, or signs of organ failure would be removed from the study and humanely euthanized according to protocol.

### 4.4. Blood and Tissue Sampling and Processing

At the end of the treatment period, ten rats were randomly selected from each experimental group, fasted for 10 h, and lightly anesthetized with tetrahydrofuran vapor to reduce handling-related stress. Animals were then euthanized by cervical dislocation in compliance with institutional and international ethical recommendations. Blood was collected from the retro-orbital venous plexus into plain collection tubes, allowed to clot at room temperature, and centrifuged to obtain serum, which was aliquoted and stored at −20 °C until biochemical assays were performed. Livers were rapidly excised, weighed, and rinsed in ice-cold 1.15% KCl to remove residual blood, and representative samples were fixed in 10% neutral buffered formalin for histopathological examination. The remaining liver tissue was homogenized in 50 mmol/L Tris–HCl buffer (pH 7.4), and the homogenate was centrifuged at 10,000× *g* for 15 min at 4 °C; the resulting supernatant was collected and stored at −80 °C for subsequent biochemical and molecular analyses.

### 4.5. Serum Biochemical Analysis

Serum total protein (TP, MET-5001), albumin (MET-5017), alanine aminotransferase (ALT, MET-5123), aspartate aminotransferase (AST, MET-5127), alkaline phosphatase (ALP, CBA-301), total bilirubin (TB, MET-5050), total cholesterol (TC, MET-5025), triglycerides (TG, MET-5027), and glucose (GLU, MET-5035) concentrations were determined using ELISA kits (Bio-Med Diagnostic, Cairo, Egypt) according to the manufacturers’ instructions. Serum globulin concentrations were determined by deducting the measured albumin value from the total serum protein concentration.

### 4.6. Oxidative Stress and Antioxidant Markers

Hepatic antioxidant status was evaluated by measuring activities of superoxide dismutase (SOD; SOD 2521), catalase (CAT; CA 2517), and glutathione peroxidase (GPx; GSH-Px 2524), and the level of reduced glutathione (GSH; GSH TA2511) using colorimetric assay kits (BioDiagnostic, Cairo, Egypt). SOD activity was determined by measuring inhibition of nitroblue tetrazolium (NBT) reduction at 560 nm; CAT activity by measuring hydrogen peroxide decomposition at 510 nm; GPx activity by measuring NADPH oxidation at 340 nm; and GSH levels per kit instructions. Lipid peroxidation (malondialdehyde, MDA; TBARS MBS268427, MyBioSource, San Diego, CA, USA) and protein carbonyl (PC; MBS8808001, MyBioSource, San Diego, CA, USA) levels were quantified by respective colorimetric assays according to the manufacturers’ protocols.

### 4.7. Assessment of Liver Signaling Proteins and Cytokines

Supernatants from liver homogenates were analyzed for high-mobility group box 1 (HMGB1; MBS729203), phosphoinositide 3-kinase (PI3K; MBS260381), mechanistic target of rapamycin (mTOR; MBS744326), tumor necrosis factor-alpha (TNF-α; CSB-E11987r, CUSABIO), transforming growth factor-beta (TGF-β; MBS260302), and interleukin-6 (IL-6; R6000B, R&D Systems, Minneapolis, MN, USA) using ELISA kits according to manufacturer instructions. Protein concentrations of tissue samples were determined by the biuret method, with target protein levels normalized and expressed per milligram of tissue protein.

### 4.8. Quantitative Analysis of Apoptosis- and Inflammation-Related Gene Expression

Liver samples were homogenized in 1 mL of QIAzol Lysis Reagent (Qiagen, Hilden, Germany) using a Tissuelyser II (Qiagen) for total RNA isolation. Phase separation was achieved by chloroform addition and centrifugation at 12,000× *g* for 15 min at 4 °C, after which the aqueous phase was collected, and RNA was precipitated with isopropanol and centrifuged at 12,000× *g* for 10 min at 4 °C. The resulting RNA pellets were washed with 75% ethanol, centrifuged at 7500× *g* for 5 min to remove residual ethanol, air-dried, and resuspended in DNase/RNase-free water (Thermo Fisher Scientific, Waltham, MA, USA). RNA yield and purity were assessed spectrophotometrically by measuring absorbance at 260 and 280 nm, and samples with an A260/A280 ratio greater than 1.9 were used for downstream applications [52].

First-strand cDNA was synthesized from total RNA using the iScript™ cDNA Synthesis Kit (Bio-Rad Laboratories, Hercules, CA, USA) following the manufacturer’s instructions. Quantitative real-time PCR was then carried out with iTaq Universal SYBR Green Supermix (Bio-Rad, Cat. No. 172-5121) on gene-specific primers targeting apoptosis- and inflammation-associated transcripts listed in Table 3, with primers synthesized by Macrogen (Seoul, Republic of Korea), reconstituted in nuclease-free water, and stored at −20 °C until use.

qRT-PCR reactions were run on a Rotor-Gene Q real-time cycler (Qiagen) under the following conditions: initial denaturation at 95 °C for 3 min, followed by 40 cycles of denaturation at 95 °C for 5 s and combined annealing/extension at 60 °C for 30 s. Each run included no-template controls to exclude reagent contamination, and amplification specificity was confirmed by melting curve analysis at the end of the run. Relative mRNA levels were calculated using the 2^−ΔΔCt^ method [53], with β-actin serving as the internal reference gene. All reactions were performed in technical triplicate.

### 4.9. Nickel Analysis in Liver Samples

To quantify hepatic nickel content, approximately 1 g of liver tissue was weighed and digested in concentrated nitric acid using a microwave digestion system. The digested samples were subsequently preconcentrated as needed and analyzed by flame atomic absorption spectrophotometry (AAS). Measurements were performed on a Perkin-Elmer 5000 spectrometer (PerkinElmer, Waltham, MA, USA) equipped with a nickel hollow-cathode lamp (4 mA) at 228.8 nm with a 0.5 nm slit width. An oxidizing flame was achieved with acetylene and air flows of 2.0 L/min and 17.0 L/min, respectively. Appropriate external calibration standards were used, and each sample was measured in triplicate to ensure analytical accuracy.

### 4.10. Liver Histopathology

Liver samples designated for histological examination were fixed in 10% neutral buffered formalin, maintaining a formalin-to-tissue volume ratio of 20:1 for 72 h. Following fixation, specimens were dehydrated via a graded ethanol sequence, cleared with xylene, and infiltrated with molten paraffin for one hour before embedding. Paraffin blocks were sectioned into 4–5 µm slices using a rotary microtome, and the sections were mounted onto glass slides and stained with hematoxylin and eosin for microscopic analysis, according to standard protocols.

For semi-quantitative assessment of hepatic steatosis and other histopathological lesions, analysis was conducted in accordance with current “American Association for the Study of Liver Diseases (AASLD) guidelines” (Supplementary Table S1). Three rats per group were randomly selected for examination, with each slide comprising three distinct liver sections. Each section was evaluated in four non-overlapping microscopic fields at 400× magnification, yielding 12 fields per animal. Lesions were graded using a semi-quantitative scale: “0 = absent, 1 = mild, 2 = moderate, 3 = severe.” The mean histological score per rat was determined by averaging the individual field scores across all examined sections.

### 4.11. Transmission Electron Microscopy (TEM)

For ultrastructural analysis, liver specimens were fixed in 2.5% glutaraldehyde prepared in 0.1 M phosphate buffer (pH 7.4) at 4 °C for 24 h, followed by postfixation in 1% osmium tetroxide for 1–2 h. Samples were then dehydrated through a graded ethanol series (50–100%), cleared in acetone, and embedded in epoxy resin. Semi-thin sections (1 µm) were stained with toluidine blue and examined by light microscopy to localize regions of interest, after which ultrathin sections (60–70 nm) were cut on an ultramicrotome, contrasted with uranyl acetate and lead citrate, and observed using a JEOL JEM-2100 transmission electron microscope (JEOL, Akishima, Tokyo, Japan) operated at 160 kV. Digital micrographs were captured and processed using dedicated TEM analysis software (DigitalMicrograph, Gatan Microscopy Suite, Gatan Inc., Pleasanton, CA, USA) and iTEM, Soft Imaging System, Olympus Soft Imaging Solutions GmbH, Münster, Germany).

### 4.12. Statistical Analysis

Data distribution was examined using the Shapiro–Wilk test for normality, and homogeneity of variances was evaluated using Levene’s test. Group differences were analyzed using one-way analysis of variance (ANOVA) in SAS (version 9.4; SAS Institute Inc., Cary, NC, USA; PROC ANOVA), followed by Tukey’s multiple-comparison test for post hoc pairwise contrasts. Data are expressed as mean ± standard deviation (SD), and differences were considered statistically significant at *p* < 0.05. All graphs were generated using “GraphPad Prism software, version 9.0 (GraphPad Software, San Diego, CA, USA)”.

## 5. Conclusions

This study demonstrates that NRG-NLPs confer superior protection against NiSO_4_-induced hepatotoxicity in rats compared with crude NRG. The nanoformulation effectively reduced oxidative stress, inflammation, apoptosis, and fibrotic changes by enhancing hepatic antioxidant defenses, suppressing inflammatory mediators, and restoring normal survival signaling. NRG-NLPs modulated key molecular pathways, including JAK/STAT, Bax/Bcl-2/Caspase-3, and downregulated hepatic HMGB1, PI3K, and mTOR, which are central to oxidative, inflammatory, and apoptotic responses. Additionally, NRG-NLPs minimized hepatic Ni accumulation and preserved near-normal liver architecture. These findings support nanoliposomal naringin as an advanced citrus-derived medicinal phytochemical and dietary bioactive strategy for the prevention and treatment of heavy metal-induced chronic liver injury, underscoring its translational relevance for chronic disease mitigation and functional food development.

## Figures and Tables

**Table 1:** Comparative evaluation of raw and nanoliposomal naringin on body weight, food and water intake, and liver-to-body weight ratio following nickel sulfate exposure in rats.

Groups	Initial Body Weight (g)	Final Body Weight (g)	Food Intake (g/100 g Body Weight/Day)	Water Intake (mL/Rat/Day)	Liver (%)
Control	174.62 ± 10.62	203.61 ± 8.12 ^a^	13.21 ± 0.64 ^a^	20.61 ± 1.14 ^a^	2.87 ± 0.39 ^b^
NRG	176.21 ± 11.26	206.94 ± 7.16 ^a^	13.16 ± 0.71 ^a^	21.16 ± 1.06 ^a^	2.97 ± 0.31 ^b^
NRG-NLPs	181.94 ± 14.82	207.49 ± 11.03 ^a^	12.94 ± 0.69 ^a^	18.61 ± 1.97 ^a^	3.01 ± 0.29 ^b^
Ni	170.46 ± 15.03	151.49 ± 7.21 ^c^	7.86 ± 0.57 ^c^	10.62 ± 1.10 ^c^	3.94 ± 0.28 ^a^
Ni + NRG	175.62 ± 13.20	171.25 ± 11.26 ^bc^	9.16 ± 0.67 ^bc^	13.21 ± 1.09 ^bc^	3.56 ± 0.31 ^ab^
Ni + NRG-NLPs	177.55 ± 12.84	184.16 ± 12.94 ^ab^	10.26 ± 0.59 ^ab^	17.25 ± 1.82 ^ab^	3.21 ± 0.22 ^b^

Values are presented as mean ± SE. Abbreviations: “NRG: Naringin (80 mg/kg body weight); NRG-NLPs: Naringin-loaded liposomal nanoparticles (80 mg/kg body weight); Ni: Nickel sulfate (20 mg/kg body weight); Ni + NRG: Combined treatment of naringin (80 mg/kg body weight) and nickel sulfate (20 mg/kg body weight); Ni + NRG-NLPs: Combined treatment of naringin-loaded liposomal nanoparticles (80 mg/kg body weight) and nickel sulfate (20 mg/kg body weight).” Significant differences (*p* < 0.05) within the same column are indicated by different superscript letters (a, b, c).

**Table 2:** Comparative evaluation of raw and nanoliposomal naringin on liver function biomarkers following nickel sulfate exposure in rats.

Parameters	Control	NRG	NRG-NLPs	Ni	Ni + NRG	Ni + NRG-NLPs
TP (g/dL)	6.62 ± 0.46 ^a^	6.78 ± 0.58 ^a^	6.93 ± 0.62 ^a^	4.11 ± 0.36 ^b^	5.12 ± 0.34 ^b^	5.67 ± 0.28 ^a^
Alb (g/dL)	3.71 ± 0.23 ^a^	3.86 ± 0.15 ^a^	3.92 ± 0.22 ^a^	2.33 ± 0.10 ^c^	2.97 ± 0.13 ^b^	3.01 ± 0.17 ^b^
Glo (g/dL)	2.91 ± 0.23 ^a^	2.92 ± 0.43 ^a^	3.01 ± 0.40 ^a^	1.78 ± 0.26 ^b^	2.15 ± 0.21 ^ab^	2.66 ± 0.11 ^a^
ALT (U/L)	41.26 ± 3.58 ^c^	40.21 ± 3.01 ^c^	40.18 ± 4.12 ^c^	78.41 ± 5.03 ^a^	62.23 ± 4.74 ^b^	51.22 ± 3.11 ^bc^
AST (U/L)	57.66 ± 3.96 ^c^	57.49 ± 4.12 ^c^	56.78 ± 5.13 ^c^	112.23 ± 6.47 ^a^	94.05 ± 4.19 ^b^	71.10 ± 4.32 ^c^
ALP (U/L)	97.51 ± 3.88 ^d^	96.24 ± 4.13 ^d^	95.45 ± 3.74 ^d^	187.44 ± 7.16 ^a^	165.23 ± 6.95 ^b^	134.59 ± 5.59 ^c^
TB (mg/dL)	0.62 ± 0.02 ^c^	0.61 ± 0.03 ^c^	0.58 ± 0.01 ^c^	1.43 ± 0.19 ^a^	1.13 ± 0.11 ^ab^	0.84 ± 0.09 ^bc^
TC (mg/dL)	81.21 ± 5.62 ^d^	80.2 ± 4.26 ^d^	79.62 ± 3.95 ^d^	161.21 ± 7.11 ^a^	132.26 ± 6.28 ^b^	102.36 ± 4.37 ^c^
TG (mg/dL)	89.61 ± 4.56 ^c^	88.74 ± 4.92 ^c^	87.69 ± 5.47 ^c^	178.62 ± 6.81 ^a^	165.23 ± 5.55 ^a^	114.25 ± 6.14 ^b^
Glu (mg/dL)	114.22 ± 5.79 ^c^	112.23 ± 4.12 ^c^	109.84 ± 3.22 ^c^	158.64 ± 4.17 ^a^	135.26 ± 4.69 ^b^	119.74 ± 3.21 ^c^

Values are presented as mean ± SE. Abbreviations: “TP: Total protein; Alb: Albumin; Glo: Globulin; ALT: Alanine aminotransferase; AST: Aspartate aminotransferase; ALP: Alkaline phosphatase; TB: Total bilirubin; TC: Total cholesterol; TG: Triglycerides; Glu: Glucose; NRG: Naringin (80 mg/kg body weight); NRG-NLPs: Naringin-loaded liposomal nanoparticles (80 mg/kg body weight); Ni: Nickel sulfate (20 mg/kg body weight); Ni + NRG: Combined treatment of naringin (80 mg/kg body weight) and nickel sulfate (20 mg/kg body weight); Ni + NRG-NLPs: Combined treatment of naringin-loaded liposomal nanoparticles (80 mg/kg body weight) and nickel sulfate (20 mg/kg body weight) (each group n = 10).” Significant differences (*p* < 0.05) within the same row are indicated by different superscript letters (a, b, c, d).

**Table 3:** Primers targeting key apoptosis- and inflammation-related genes.

Gene	Sequences (5′-3′)	Accession No.	Length (bp)
*Bax*	F: TTTCATCCAGGATCGAGCAG R: AATCATCCTCTGCAGCTCCA	NM_017059.2	154
*BCL-2*	F: TCGCGACTTTGCAGAGATGT R: CAATCCTCCCCCAGTTCACC	NM_016993.2	116
*Caspase-3*	F: ACTGGAATGTCAGCTCGCAA R: GCAGTAGTCGCCTCTGAAGA	NM_012922.2	270
*JAK*	F: AGCTCCTCTCCTTGACGACT R: CACGCACTTCGGTAAGAAC	NM_012859.3	150
*STAT*	F: AGCAATACCATTGAC CTGCC R: TTTGGCTGCTTAAGGGGTGG	NM_012448.2	130
*Nrf2*	F: TTTGTAGATGACCATGAGTCGC R: TCCTGCCAAACTTGCTCCAT		161
*HO-1*	F: ATGTCCCAGGATTTGTCCGA R: ATGGTACAAGGAGGCCATCA		144
*NFκB*	F: AGTCCCGCCCCTTCTAAAAC R: CAATGGCCTCTGTGTAGCCC	NM_001276711.1	106
*TNF-α*	F: CTCGAGTGACAAGCCCGTAG R: ATCTGCTGGTACCACCAGTT	NM_012675.3	139
*IL-6*	F: AGCGATGATGCACTGTCAGA R: GGAACTCCAGAAGACCAGAGC	NM_012589.2	127
*β-Actin*	F: CAGCCTTCCTTCTTGGGTATG R: AGCTCAGTAACAGTCCGCCT	NM_031144.3	360

“*Bax*: Bcl-2-associated X protein; *Bcl-2*: B-cell lymphoma 2; *Caspase-3*: Cysteine-aspartic protease 3; *JAK*: Janus Kinase; *STAT*: Signal Transducer and Activator of Transcription; *Nrf2*: Nuclear factor erythroid 2-related factor 2; *HO-1*: Heme oxygenase-1; *NF-κB*: Nuclear Factor kappa-light-chain-enhancer of activated B cells; *TNF-α*: Tumor Necrosis Factor-alpha; *IL-6*: Interleukin-6.”

## Data Availability

The original contributions presented in this study are included in the article and Supplementary Materials. Further inquiries can be directed to the corresponding author.

## Supplementary Materials

The following supporting information can be downloaded at: https://www.mdpi.com/article/10.3390/ph19010051/s1, Table S1: Scoring Scheme for Liver Tissue Histopathological Changes.

## Abbreviations

The following abbreviations are used in this manuscript:

AS	Atomic absorption spectrophotometry
Alb	Albumin
ALP	Alkaline phosphatase
ALT	Alanine aminotransferase
ANOVA	Analysis of variance
AST	Aspartate aminotransferase
Bax	Bcl-2-associated X protein
Bcl-2	B-cell lymphoma 2
CAT	Catalase
DLS	Dynamic light scattering
ELISA	Enzyme-linked immunosorbent assay
FTIR	Fourier-transform infrared spectroscopy
Glu	Glucose
Glo	Globulin
GPx	Glutathione peroxidase
GSH	Reduced glutathione
H&E	Hematoxylin and eosin
HMGB1	High-mobility group box 1
HO-1	Heme oxygenase-1
IL-6	Interleukin-6
JAK	Janus kinase
LE	Loading efficiency
mTOR	Mechanistic target of rapamycin
MDA	Malondialdehyde
NF-κB	Nuclear factor kappa-light-chain-enhancer of activated B cells
Ni	Nickel
NRG	Naringin
NRG-NLPs	Naringin-loaded nanoliposomes
NiSO_4_	Nickel sulfate
Nrf2	Nuclear Factor Erythroid 2–Related Factor 2
PDI	Polydispersity index
PBS	Phosphate-buffered saline
PC	Protein carbonyl
PI3K	Phosphoinositide 3-kinase
rER	Rough endoplasmic reticulum
ROS	Reactive oxygen species
SD	Standard deviation
SOD	Superoxide dismutase
STAT	Signal transducer and activator of transcription
TB	Total bilirubin
TC	Total cholesterol
TGF-β	Transforming growth factor-beta
TEM	Transmission electron microscopy
TG	Triglycerides
TNF-α	Tumor necrosis factor-alpha
TP	Total protein
ZP	Zeta potential

## References

[1] Buxton S., Garman E., Heim K.E., Lyons-Darden T., Schlekat C.E., Taylor M.D., Oller A.R. (2019). Concise Review of Nickel Human Health Toxicology and Ecotoxicology. Inorganics.

[2] Iqbal S., Jabeen F., Peng C., Shah M.A., Ijaz M.U., Rasul A., Ali S., Rauf A., Batiha G.E., Kłodzińska E. (2021). Nickel nanoparticles induce hepatotoxicity via oxidative and nitrative stress-mediated apoptosis and inflammation. Toxicol. Ind. Health.

[3] Genchi G., Carocci A., Lauria G., Sinicropi M.S., Catalano A. (2020). Nickel: Human Health and Environmental Toxicology. Int. J. Environ. Res. Public Health.

[4] Lala V., Zubair M., Minter D. (2023). Liver function tests. StatPearls [Internet].

[5] Vardar Acar N., Özgül R.K. (2023). The bridge between cell survival and cell death: Reactive oxygen species-mediated cellular stress. EXCLI J..

[6] Boroun R., Dehagi B., Ahmadizadeh M. (2020). Protective effect of vitamin E on nickel sulfate-induced renal dysfunction in rats. J. Ren. Inj. Prev..

[7] Abdulqadir S., Aziz F. (2019). Hepatotoxicity of nickel nanoparticles in rats. Indian J. Anim. Res..

[8] Juan C.A., Pérez de la Lastra J.M., Plou F.J., Pérez-Lebeña E. (2021). The Chemistry of Reactive Oxygen Species (ROS) Revisited: Outlining Their Role in Biological Macromolecules (DNA, Lipids and Proteins) and Induced Pathologies. Int. J. Mol. Sci..

[9] Zhong C., Hou S., Guo J., Zhao J., Wang J., Fang Y., Liu H., Ding H., Ma X., Lyu W. (2025). Nickel exposure induced endoplasmic reticulum stress by disturbing calcium homeostasis and triggered mitochondrial autophagy in domestic animal oocytes. Ecotoxicol. Environ. Saf..

[10] Zahra M., Abrahamse H., George B.P. (2024). Flavonoids: Antioxidant Powerhouses and Their Role in Nanomedicine. Antioxidants.

[11] Al-Ghamdi N., Virk P., Hendi A., Awad M., Elobeid M. (2021). Antioxidant potential of bulk and nanoparticles of naringenin against cadmium-induced oxidative stress in Nile tilapia, Oreochromis niloticus. Green Process. Synth..

[12] Bodakowska-Boczniewicz J., Garncarek Z. (2025). Use of Naringinase to Modify the Sensory Quality of Foods and Increase the Bioavailability of Flavonoids: A Systematic Review. Molecules.

[13] Flores-Peña R., Monroy-Ramirez H.C., Caloca-Camarena F., Arceo-Orozco S., Salto-Sevilla J.A., Galicia-Moreno M., Armendariz-Borunda J. (2025). Naringin and Naringenin in Liver Health: A Review of Molecular and Epigenetic Mechanisms and Emerging Therapeutic Strategies. Antioxidants.

[14] Stabrauskiene J., Kopustinskiene D.M., Lazauskas R., Bernatoniene J. (2022). Naringin and Naringenin: Their Mechanisms of Action and the Potential Anticancer Activities. Biomedicines.

[15] Ravetti S., Garro A.G., Gaitán A., Murature M., Galiano M., Brignone S.G., Palma S.D. (2023). Naringin: Nanotechnological Strategies for Potential Pharmaceutical Applications. Pharmaceutics.

[16] Ghanty S., Ganguly A., Nanda S., Mandi M., Das K., Biswas G., Maitra P., Khatun N., Rajak P. (2025). Antioxidant and Pro-oxidant properties of naringenin: Unveiling the biphasic impacts on model, Drosophila melanogaster. Food Chem. Adv..

[17] García-Morales J., Fimbres-Olivarría D., González-Vega R.I., Bernal-Mercado A.T., Aubourg-Martínez S.P., López-Gastélum K.A., Robles-García M.Á., de Jesús Ornelas-Paz J., Ruiz-Cruz S., Del-Toro-Sánchez C.L. (2025). Nanoliposomes as Effective Vehicles of Antioxidant Compounds in Food and Health. Int. J. Mol. Sci..

[18] Yu S., Liu F., Wang C., Zhang J., Zhu A., Zou L., Han A., Li J., Chang X., Sun Y. (2018). Role of oxidative stress in liver toxicity induced by nickel oxide nanoparticles in rats. Mol. Med. Rep..

[19] Sun T., Kang Y., Liu J., Zhang Y., Ou L., Liu X., Lai R., Shao L. (2021). Nanomaterials and hepatic disease: Toxicokinetics, disease types, intrinsic mechanisms, liver susceptibility, and influencing factors. J. Nanobiotechnol..

[20] Lee J., Kim J., Lee R., Lee E., Choi T.G., Lee A.S., Yoon Y.I., Park G.C., Namgoong J.M., Lee S.G. (2022). Therapeutic strategies for liver diseases based on redox control systems. Biomed. Pharmacother..

[21] Owonikoko M.W., Emikpe B.O., Olaleye S.B. (2021). Standardized experimental model for cement dust exposure; tissue heavy metal bioaccumulation and pulmonary pathological changes in rats. Toxicol. Rep..

[22] Lazic S.E., Semenova E., Williams D.P. (2020). Determining organ weight toxicity with Bayesian causal models: Improving on the analysis of relative organ weights. Sci. Rep..

[23] Abouzeinab N.S., Kahil N., Fakhruddin N., Awad R., Khalil M.I. (2023). Intraperitoneal hepato-renal toxicity of zinc oxide and nickel oxide nanoparticles in male rats: Biochemical, hematological and histopathological studies. EXCLI J..

[24] Sun H., Qiang Z., Tang W., Shu Y., Zou Z., Zhang G. (2007). Metallothionein expression induced by nickel accumulation in the midgut of Spodoptera litura Fabricius larvae exposed to nickel. Chin. Sci. Bull..

[25] Thakur S., Kumar V., Das R., Sharma V., Mehta D.K. (2024). Biomarkers of Hepatic Toxicity: An Overview. Curr. Ther. Res. Clin. Exp..

[26] Meseguer E.S., Elizalde M.U., Borobia A.M., Ramírez E. (2021). Valproic Acid-Induced Liver Injury: A Case-Control Study from a Prospective Pharmacovigilance Program in a Tertiary Hospital. J. Clin. Med..

[27] Conde de la Rosa L., Goicoechea L., Torres S., Garcia-Ruiz C., Fernandez-Checa J.C. (2022). Role of Oxidative Stress in Liver Disorders. Livers.

[28] Zhang Q., Chang X., Wang X., Zhan H., Gao Q., Yang M., Liu H., Li S., Sun Y. (2021). A metabolomic-based study on disturbance of bile acids metabolism induced by intratracheal instillation of nickel oxide nanoparticles in rats. Toxicol. Res..

[29] Raja Kumar S., Mohd Ramli E.S., Abdul Nasir N.A., Ismail N.H.M., Mohd Fahami N.A. (2019). Preventive Effect of Naringin on Metabolic Syndrome and Its Mechanism of Action: A Systematic Review. Evid.-Based Complement. Altern. Med. eCAM.

[30] Shilpa V.S., Shams R., Dash K.K., Pandey V.K., Dar A.H., Ayaz Mukarram S., Harsányi E., Kovács B. (2023). Phytochemical Properties, Extraction, and Pharmacological Benefits of Naringin: A Review. Molecules.

[31] Rashmi R., Bojan Magesh S., Mohanram Ramkumar K., Suryanarayanan S., Venkata SubbaRao M. (2018). Antioxidant Potential of Naringenin Helps to Protect Liver Tissue from Streptozotocin-Induced Damage. Rep. Biochem. Mol. Biol..

[32] Jiang H., Zhang M., Lin X., Zheng X., Qi H., Chen J., Zeng X., Bai W., Xiao G. (2023). Biological Activities and Solubilization Methodologies of Naringin. Foods.

[33] Pari L., Amudha K. (2011). Hepatoprotective role of naringin on nickel-induced toxicity in male Wistar rats. Eur. J. Pharmacol..

[34] Abdelhamid F.M., Mahgoub H.A., Ateya A.I. (2020). Ameliorative effect of curcumin against lead acetate-induced hemato-biochemical alterations, hepatotoxicity, and testicular oxidative damage in rats. Environ. Sci. Pollut. Res. Int..

[35] Lu R., Yu R.J., Yang C., Wang Q., Xuan Y., Wang Z., He Z., Xu Y., Kou L., Zhao Y.Z. (2022). Evaluation of the hepatoprotective effect of naringenin loaded nanoparticles against acetaminophen overdose toxicity. Drug Deliv..

[36] Atta R., Arafat H.E.K., Khalil I.A., Ali D.A., Abd El-Fadeal N.M., Kattan S.W., Alelwani W., Fawzy M.S., Mansour M.F. (2025). Enhanced hepatoprotective efficacy of quercetin nanoparticles versus free quercetin against acrylamide-induced hepatotoxicity through modulation of MAPK/NF-κB/NLRP3 signaling pathways and molecular docking validation. Tissue Cell.

[37] Shaalan A.A.M., Elmorsy E.M., Embaby E.M., Alfawaz M., Aly N.M., Shams A.S., Fawzy M.S., Hosny N. (2025). Antitumor, Antioxidant, and Hepatoprotective Effects of Grape Seed Oil Nanoemulsion as a Dietary Phytochemical Intervention in Ehrlich Solid Tumors. Nutrients.

[38] Chang X., Liu F., Tian M., Zhao H., Han A., Sun Y. (2017). Nickel oxide nanoparticles induce hepatocyte apoptosis via activating endoplasmic reticulum stress pathways in rats. Environ. Toxicol..

[39] Liu T., Zhang L., Joo D., Sun S.C. (2017). NF-κB signaling in inflammation. Signal Transduct. Target. Ther..

[40] Li M., Zhang X., Wang B., Xu X., Wu X., Guo M., Wang F. (2018). Effect of JAK2/STAT3 signaling pathway on liver injury associated with severe acute pancreatitis in rats. Exp. Ther. Med..

[41] Tian L.Y., Smit D.J., Jücker M. (2023). The Role of PI3K/AKT/mTOR Signaling in Hepatocellular Carcinoma Metabolism. Int. J. Mol. Sci..

[42] Chen R., Kang R., Tang D. (2022). The mechanism of HMGB1 secretion and release. Exp. Mol. Med..

[43] Kim K.K., Sheppard D., Chapman H.A. (2018). TGF-β1 Signaling and Tissue Fibrosis. Cold Spring Harb. Perspect. Biol..

[44] Dewidar B., Meyer C., Dooley S., Meindl-Beinker A.N. (2019). TGF-β in Hepatic Stellate Cell Activation and Liver Fibrogenesis-Updated 2019. Cells.

[45] Ortiz C., Schierwagen R., Schaefer L., Klein S., Trepat X., Trebicka J. (2021). Extracellular Matrix Remodeling in Chronic Liver Disease. Curr. Tissue Microenviron. Rep..

[46] Wang W., Gao Y., Chen Y., Cheng M., Sang Y., Wei L., Dai R., Wang Y., Zhang L. (2025). TGF-β inhibitors: The future for prevention and treatment of liver fibrosis?. Front. Immunol..

[47] Mansour L.A.H., Elshopakey G.E., Abdelhamid F.M., Albukhari T.A., Almehmadi S.J., Refaat B., El-Boshy M., Risha E.F. (2023). Hepatoprotective and Neuroprotective Effects of Naringenin against Lead-Induced Oxidative Stress, Inflammation, and Apoptosis in Rats. Biomedicines.

[48] Naraki K., Rezaee R., Karimi G. (2021). A review on the protective effects of naringenin against natural and chemical toxic agents. Phytother. Res. PTR.

[49] Yan S., Zhang G., Luo W., Xu M., Peng R., Du Z., Liu Y., Bai Z., Xiao X., Qin S. (2024). PROTAC technology: From drug development to probe technology for target deconvolution. Eur. J. Med. Chem..

[50] Tu Y., Dai G., Chen Y., Tan L., Liu H., Chen M. (2025). Emerging Target Discovery Strategies Drive the Decoding of Therapeutic Power of Natural Products and Further Drug Development: A Case Study of Celastrol. Exploration.

[51] Zhang X., Hartmann P. (2023). How to calculate sample size in animal and human studies. Front. Med..

[52] Emaus M.N., Varona M., Eitzmann D.R., Hsieh S.-A., Zeger V.R., Anderson J.L. (2020). Nucleic acid extraction: Fundamentals of sample preparation methodologies, current advancements, and future endeavors. TrAC Trends Anal. Chem..

[53] Livak K.J., Schmittgen T.D. (2001). Analysis of relative gene expression data using real-time quantitative PCR and the 2(-Delta Delta C(T)) Method. Methods.

