# Supplementary material for: Hepatoprotection by Naringin Nanoliposomes Against Nickel Toxicity Involves Antioxidant Reinforcement and Modulation of Nrf2, NF-κB, PI3K/mTOR, JAK/STAT, and Apoptotic Pathways
Source: Pharmaceuticals (Basel). 2025 Dec 25;19(1):51. doi: 10.3390/ph19010051 (PMC12844761; doi:10.3390/ph19010051)
Supplement: Supplementary file 1 [file pharmaceuticals-19-00051-s001.zip › pharmaceuticals-4052856-supplementary.pdf]

Hepatoprotection by Naringin Nanoliposomes Against Nickel Toxicity Involves Antioxidant Reinforcement and Modulation of Nrf2, NF-κB, PI3K/mTOR, JAK/STAT, and Apoptotic Pathways

Table S1. Scoring Scheme for Liver Tissue Histopathological Changes.

**Score**	Composite Liver Assessment Score
0 (none)	No pathological changes
1 (mild)	Hepatic tissue showed mild-to-rare hepatocyte degeneration and necrosis, with minimal inflammation and occasional vascular congestion.
2 (moderate)	Hepatic tissue showed moderate vacuolar degeneration, multifocal hepatocellular necrosis, associated inflammation, and minor vascular congestion.
3 (severe)	Hepatic tissue showed severe degeneration, extensive necrosis, pronounced inflammation, and moderate-to-marked vascular congestion.
